# P21 - Evaluation of modifications in SCORMA Index and grading in the follow-up of a pediatric population with mastocytosis

**DOI:** 10.1186/2045-7022-4-S1-P76

**Published:** 2014-02-28

**Authors:** Andrea Del Mastro, Angelica Petraroli, Diomira Magliacane, Veronica Squeglia, Carmela Gravante, Alessandro Barbarino, Giuseppe Spadaro, Gianni Marone

**Affiliations:** 1 Division of Allergy and Clinical Immunology, University of Naples Federico II, Naples, Italy; 2 Division of Allergy and Respiratory Disease, Hospital of Battipaglia, Salerno, Italy; 3 Division of Preventive Medicine, University of Naples Federico II, Italy

## Background

Mastocytosis is a rare disease affecting both children and adults. It is characterized by accumulation of mast-cells in the skin and/or other tissues so that two main variants are distinguished: cutaneous (CM) and systemic (SM) mastocytosis. Most pediatric patients have primarily CM and complete remission will develop in a considerable number of patients during puberty. Less frequently, skin lesions persist and these patients are often diagnosed with SM in adulthood.

Our study investigates the correlation between SCORMA (SCORing MAstocytosis) Index (SC-I), a scoring system that assesses the clinical extent and intensity of cutaneous mastocytosis, and Grading system, that evaluates skin-specific symptoms and treatment status, in the follow-up of pediatric patients with mastocytosis. Furthermore, we evaluate the role of SC-I modifications in the assessment of the severity of disease.

## Methods

Twenty-two patients aged less than 12 years were enrolled from our centre. SC-I and Grading were assessed for each patient at first visit and after 2 years, with determination of Grading modifications and calculation of differences in absolute value of SC-I (ΔSC-I). Data were analyzed using SPSS software. Pearson’s correlation coefficient (ρ) was used to assess the relationship between SC-I and Grading at first visit (ρ_1_) and after 2 years (ρ_2_). Chi-square test was performed to evaluate the association of ΔSC-I with Grading modifications, by dividing patients with SC-I changed (ΔSC-I > 10) and unchanged (ΔSC-I ≤ 10) in two groups (Grading modified and unmodified).

## Results

We found ρ_1_= 0, 47 (p < 0,05) and ρ_2_ =0,36 (p < 0,05), while there was no statistical difference in ΔSC-I in the two groups (p < 0,05).

## Conclusion

According to our data, in pediatric patients with mastocytosis SC-I and Grading show a moderate correlation at first visit which weakens not significantly at the subsequent assessment after 2 years. Moreover, SC-I modifications (ΔSC-I) seem to be independent from Grading modifications and, as a consequence, from the clinical evolution of disease.

